# Supplementary material for: The Systems Biology Research Tool: evolvable open-source software
Source: BMC Syst Biol. 2008 Jun 29;2:55. doi: 10.1186/1752-0509-2-55 (PMC2446383; doi:10.1186/1752-0509-2-55)
Supplement: Additional file 1 — SBRT Archive. An archive of the current version of the Systems Biology Research Tool. [file 1752-0509-2-55-S1.zip › sbrt-1.4.0/doc/developers_guide/api/index-files/index-20.html]

T-Index


|  |  |  |
| --- | --- | --- |
| **Overview** \| Package \| Class \| Use \| **Tree** \| **Deprecated** \| **Index** \| **Help** | |  |
| **PREV LETTER**   **NEXT LETTER** | **FRAMES**    **NO FRAMES**     **All Classes** |  |


A B C D E F G H I J K L M N O P Q R S T U V W X Y Z 

---


## **T**

**TabListFormat**<E> - Class in sbrt.shell.text: This class is used to define a format for tab-delimited lists. **TabListFormat(SimpleFormat<E>)** - Constructor for class sbrt.shell.text.TabListFormat: Constructs a new tab-delimited list format with the provided element format. **TabListFormat(Formatter<E>, Parser<E>)** - Constructor for class sbrt.shell.text.TabListFormat: Constructs a new tab-delimited list format with the provided element format. **TabSetFormat**<E> - Class in sbrt.shell.text: This class is used to define a format for tab-delimited sets. **TabSetFormat(SimpleFormat<E>)** - Constructor for class sbrt.shell.text.TabSetFormat: Constructs a new tab-delimited set format with the provided element format. **TabSetFormat(Formatter<E>, Parser<E>)** - Constructor for class sbrt.shell.text.TabSetFormat: Constructs a new tab-delimited set format with the provided element format. **TAU\_A** - Static variable in class sbrt.shell.mng.stat.KendallTauManager: The value used to denote a Kendall's Tau_A_ correlation coefficient. **TAU\_B** - Static variable in class sbrt.shell.mng.stat.KendallTauManager: The value used to denote a Kendall's Tau_B_ correlation coefficient. **TauResult** - Class in sbrt.kernel.math.stat.comp: This class is used to store the results of a correlation computation. **TauResult(double, double, int)** - Constructor for class sbrt.kernel.math.stat.comp.TauResult: Constructs a new correlation results. **Taxicab** - Class in sbrt.kernel.math.geom.comp: This class is used to compute Taxicab distances. **Taxicab()** - Constructor for class sbrt.kernel.math.geom.comp.Taxicab: **terms()** - Method in class sbrt.kernel.math.HashLinearComb: Returns the number of terms contained in this linear combination. **terms()** - Method in interface sbrt.kernel.math.Polynomial: Returns the number of terms contained in this polynomial. **TEXT** - Static variable in class sbrt.shell.io.FileFormat: Used to denote a plain-text file. **TextAreaOutputStream** - Class in sbrt.shell.gui: An output stream that writes its output to a javax.swing.JTextArea control. **TextAreaOutputStream(JTextArea)** - Constructor for class sbrt.shell.gui.TextAreaOutputStream: Creates a new instance of TextAreaOutputStream which writes to the specified instance of javax.swing.JTextArea control. **TextFileLineIterator**<E> - Class in sbrt.shell.io: This class is used to iterate over the lines of an input file. **TextFileLineIterator(TextInputFile<E>)** - Constructor for class sbrt.shell.io.TextFileLineIterator: Constructs a new text file line iterator using the provided text input file. **TextInputFile**<E> - Class in sbrt.shell.io: This class is used to read input files containing text. **TextInputFile(String, Parser<E>)** - Constructor for class sbrt.shell.io.TextInputFile: Constructs a new text input file using the provided arguments. **TextInputFile(String, FileFormat, Parser<E>)** - Constructor for class sbrt.shell.io.TextInputFile: Constructs a new text input file using the provided arguments. **TextInputFile(String, FileFormat, Parser<E>, Collection<Character>)** - Constructor for class sbrt.shell.io.TextInputFile: Constructs a new text input file using the provided arguments. **TextOutputFile**<T> - Class in sbrt.shell.io: This class is used to represent output files containing plain text. **TextOutputFile(String, Formatter<T>)** - Constructor for class sbrt.shell.io.TextOutputFile: Constructs a new text output file using the provided arguments. **TextOutputFile(String, FileFormat, Formatter<T>)** - Constructor for class sbrt.shell.io.TextOutputFile: Constructs a new text output file using the provided arguments. **TextPaneGui** - Class in sbrt.shell.gui: This class is used to create a GUI for the SBRT. **TextPaneGui()** - Constructor for class sbrt.shell.gui.TextPaneGui: Constructs a new text pane GUI for the SBRT. **TextPaneOutputStream** - Class in sbrt.shell.gui: An output stream that writes its output to a javax.swing.JTextArea control. **TextPaneOutputStream(JTextPane, String)** - Constructor for class sbrt.shell.gui.TextPaneOutputStream: Creates a new instance of TextAreaOutputStream which writes to the specified instance of javax.swing.JTextArea control. **TILDE** - Static variable in class sbrt.shell.text.SpecialChars: ~ **toArray()** - Method in class sbrt.kernel.math.graph\_theory.Path: Returns an array representation of this path. **toArray()** - Method in class sbrt.kernel.util.UnmodifiableCollection: See Collection.toArray(). **toArray(T[])** - Method in class sbrt.kernel.util.UnmodifiableCollection: See Collection.toArray(Object[]). **toArray()** - Method in class sbrt.kernel.util.UnmodifiableList: See List.toArray(). **toArray(T[])** - Method in class sbrt.kernel.util.UnmodifiableList: See List.toArray(Object[]). **toArray()** - Method in class sbrt.kernel.util.UnmodifiableSet: See Set.toArray(). **toArray(T[])** - Method in class sbrt.kernel.util.UnmodifiableSet: See Set.toArray(Object[]). **toLinearCombs()** - Method in class sbrt.kernel.math.algebra.AbstractSmdMatrix: Returns this matrix as a list of linear combinations. **toLinearCombs()** - Method in interface sbrt.kernel.math.algebra.DoubleMatrix: Returns this matrix as a list of linear combinations. **toLinearCombs()** - Method in class sbrt.kernel.math.algebra.DoubleMatrixWrap: Returns this matrix as a list of linear combinations. **toList()** - Method in class sbrt.kernel.math.graph\_theory.Path: Returns a list representation of this path. **toMap()** - Method in class sbrt.kernel.math.algebra.HashLinearSystemSolution: Returns this solution as a map. **toMap()** - Method in interface sbrt.kernel.math.algebra.LinearSystemSolution: Returns this solution as a map. **toMap()** - Method in class sbrt.shell.io.ProcessInputFile: Returns the information contained in this file as a map. **toMap()** - Method in interface sbrt.shell.mng.ManagerInput: Returns the information contained in this input as a map. **toMap()** - Method in class sbrt.shell.mng.MapManagerInput: Returns the information contained in this input as a map. **toString()** - Method in class sbrt.ext.cplex.CplexSolver: Returns the name of this program solver. **toString()** - Method in class sbrt.ext.R.stat.RCorrelator.Result: Returns a string representation of this result. **toString()** - Method in class sbrt.kernel.fba.AbstractRxn: Returns an appropriate string representation of this reaction. **toString()** - Method in interface sbrt.kernel.fba.ChemRxn: Returns an appropriate string representation of this chemical reaction. **toString()** - Method in class sbrt.kernel.fba.IrrevRxn: Returns an appropriate string representation of this irreversible reaction. **toString()** - Method in class sbrt.kernel.fba.proc.FbaProcess: Returns a string representation of this experiment. **toString()** - Method in class sbrt.kernel.fba.ReversibleRxn: Returns an appropriate string representation of this reversible reaction. **toString()** - Method in class sbrt.kernel.fba.SinkRxn: Returns a string representation of this sink reaction. **toString()** - Method in class sbrt.kernel.fba.SourceRxn: Returns a string representation of this source reaction. **toString()** - Method in class sbrt.kernel.math.AbstractLinearComb.Term: Returns an appropriate string representation of this term. **toString()** - Method in class sbrt.kernel.math.AbstractPolynomial: Returns a string representation of this polynomial. **toString()** - Method in class sbrt.kernel.math.algebra.AbstractMdMatrix: Returns a string representation of this matrix. **toString()** - Method in interface sbrt.kernel.math.algebra.Matrix: Returns a string representation of this matrix. **toString()** - Method in class sbrt.kernel.math.algebra.MatrixWrap: Returns a string representation of this matrix. **toString()** - Method in class sbrt.kernel.math.geom.ParallelHyperplanes: Returns a string representation of this pair of bounding parallel hyperplanes. **toString()** - Method in class sbrt.kernel.math.graph\_theory.DirectedGraph.Edge: Returns an appropriate string representation of this edge. **toString()** - Method in class sbrt.kernel.math.graph\_theory.Path: Returns a string representation of this path. **toString()** - Method in class sbrt.kernel.math.Interval: Returns a string representation of this interval. **toString()** - Method in interface sbrt.kernel.math.LinearComb: Returns an appropriate string representation of this linear combination. **toString()** - Method in interface sbrt.kernel.math.MathExpr: Returns an appropriate string representation of this mathematical expression. **toString()** - Method in enum sbrt.kernel.math.optimization.OptimizationSense: Returns an appropriate string representation of this optimization sense. **toString()** - Method in enum sbrt.kernel.math.optimization.ProgramSolverStatus: Returns an appropriate string representation of this program solver status. **toString()** - Method in interface sbrt.kernel.math.Polynomial.Term: Returns an appropriate string representation of this term. **toString()** - Method in interface sbrt.kernel.math.Polynomial: Returns an appropriate string representation of this polynomial. **toString()** - Method in class sbrt.kernel.math.stat.comp.MannWhitneyResults: Returns a string represenation of these results. **toString()** - Method in class sbrt.kernel.math.stat.comp.TauResult: Returns a string represenation of these correlation results. **toString()** - Method in class sbrt.kernel.util.And: **toString()** - Method in class sbrt.kernel.util.Or: **toString()** - Method in class sbrt.kernel.util.UnmodifiableCollection: Returns `toString()` on the underlying collection. **toString()** - Method in class sbrt.kernel.util.UnmodifiableList: Returns `toString()` on the underlying list. **toString()** - Method in class sbrt.kernel.util.UnmodifiableMap: Returns `toString()` on the underlying map. **toString()** - Method in class sbrt.kernel.util.UnmodifiableSet: Returns `toString()` on the underlying set. **toString()** - Method in class sbrt.shell.io.FileFormat: Returns a string representation of this file format. **toString()** - Method in class sbrt.shell.io.ProcessInputFile: Returns a string representation of the information contained in this file. **total()** - Method in class sbrt.kernel.fba.FluxConstraints: Returns the total number of elements contained in this set of constraints. **translate(String, String, String, PrintWriter)** - Method in class sbrt.shell.proc.fba.BiggSbmlTranslater: Translates an SBML file into an FBA Reaction File and an FBA Catalyst File. **translate(String, String, String, String, PrintWriter)** - Method in class sbrt.shell.proc.fba.PalssonSbmlTranslater: Translates an SBML file into an FBA Reaction File and two FBA Catalyst Files. **translate(String, String)** - Method in class sbrt.shell.proc.fba.SimpleRxnFileTranslator: Translates a simple reaction file into an FBA Reaction File. **translate(String, String, PrintWriter)** - Method in class sbrt.shell.proc.fba.SimpleRxnFileTranslator: Translates a simple reaction file into an FBA Reaction File. **transpose(Matrix<R, C, E>, MatrixBuilder<? super C, ? super R, E>)** - Method in class sbrt.kernel.math.algebra.MatrixTransposer: Fills `emptyMatrix` with the transpose of `matrix`. **TreeMatrix**<R extends java.lang.Comparable<? super R>,C extends java.lang.Comparable<? super C>> - Class in sbrt.kernel.math.algebra: This class is a `java.util.TreeMap` based implementation of `SmdMatrix`. **TreeMatrix()** - Constructor for class sbrt.kernel.math.algebra.TreeMatrix: Constructs an emtpy `TreeMatrix`. **TrueFalseFormat** - Class in sbrt.shell.text: This class is used to define a format for true/false values. **TrueFalseFormat()** - Constructor for class sbrt.shell.text.TrueFalseFormat: Constructs a new true/false format object.

---


|  |  |  |
| --- | --- | --- |
| **Overview** \| Package \| Class \| Use \| **Tree** \| **Deprecated** \| **Index** \| **Help** | |  |
| **PREV LETTER**   **NEXT LETTER** | **FRAMES**    **NO FRAMES**     **All Classes** |  |


A B C D E F G H I J K L M N O P Q R S T U V W X Y Z 

---
